# Synthesis of pyridinium-based ionic liquids and their application to improve *Candida rugosa* lipase stability in a methanol-water solvent system

**DOI:** 10.55730/1300-0527.3539

**Published:** 2022-10-31

**Authors:** Oktavianus HENDRA CIPTA, Anita ALNI, Rukman HERTADI

**Affiliations:** Department of Chemistry, Bandung Institute of Technology, Bandung, Indonesia

**Keywords:** *Candida rugosa* lipase, hydrolysis, ionic liquids, pyridinium, methanol

## Abstract

This paper studied the effect of pyridinium-based ionic liquids as cosolvents in a methanol-water solvent system on the hydrolytic activity of *Candida rugosa* lipase. These ionic liquids were successfully synthesized using imidazolium-based ionic liquid synthesizing methods with a certain adjustment. The hydrolytic activity of *Candida rugosa* lipase was analyzed using 4-nitrophenol acetate (pNPA) and 4-nitrophenol palmitate (pNPP) as substrates. The addition of ionic liquids had no significant effect on the hydrolytic activity of lipase in a water solvent, and it had a greater effect in methanol. The addition of [C6Py] Br ionic liquid as a methanol cosolvent (methanol: ionic liquid, 10:5) could increase the hydrolytic activity of lipase. The use of ionic liquid as a cosolvent could increase the hydrolytic activity of lipase by about 15.61% while using pNPP as a substrate in the methanol system. A molecular dynamics study for the interaction between lipase and ionic liquids supported the experimental results. The ionic liquid using bromide as an anion provided more stability on lipase conformation. It tends to form the short-range interaction between the lipase and bromide anion.

## 1. Introduction

Lipase (EC 3.1.1.3) is a universal enzyme that belongs to a group of hydrolase enzymes [1]. It is commonly used as a biocatalyst for various reactions, such as hydrolysis, transesterification, and esterification [2]. Thus, it becomes the third most used enzyme in industry, such as agrochemicals, pharmaceuticals, surfactants, and food [3, 4]. However, the enzymatic reaction is preferable since it is safer than chemical reactions, and it will follow safety, health, and environmental regulations [1].

Lipase has better hydrolytic activity in water than organic solvents [5] because organic solvents can denaturize lipase and cause conformational changes, disrupting their function and catalytic activity [6]. However, several substrates are insoluble in a water solvent, so the organic solvent should be used to run the reaction. In contrast, due to green chemistry, organic solvents should be avoided because of their toxicity, volatility, and flammable properties. Therefore, it is necessary to substitute the organic solvent to conduct the reaction.

One of the alternative solvents to replace organic solvents is ionic liquids. Compared to organic solvents, ionic liquids have various advantages, such as nonvolatility, nonflammability, and recyclability with tunable physical properties [7]. It is also able to increase thermal stability, selectivity, and enantioselectivity [8]. Thus, it is widely used in various fields, such as anticorrosion materials [9], vitamin base ingredients [10], amyloid insulin recycling [10], and also as media for enzymatic reactions [11].

Ionic liquids as a reaction media for lipase have been used by Lv et al. (2016) to evaluate the effect of liquid ionic properties on lipase activity [12]. It presented that the longer the alkyl chain of the ionic liquid, the lesser the concentration of ionic liquids required. In 2013, Na et al. (2013) conducted research on the effect of anion differences in ionic liquids on the hydrolytic activity of lipases [13]. The results showed that the use of bromide as the anion of ionic liquid could significantly increase the hydrolytic activity of lipase compared to chloride, tetrafluoroborate, and hexafluorophosphate as an anion.

Previous studies have reported research on the application of ionic liquid as a reaction media for lipase, including the use of imidazolium-based ionic liquid [14], phosphonium-based ionic liquids [15], ammonium-based ionic liquids [16], and tropin-amino acid ILs [17] successfully. To the best of the authors’ knowledge, there is not any application of pyridinium-based ionic liquids for lipase-catalyzed reactions before. A previous study has concluded that pyridinium-based ionic liquids have the best thermal stability compared with other ionic liquids [18]. Moreover, pyridinium-based ionic liquids are more environmentally friendly than other ionic liquids since they are degradable through electron degradation [19]. Pyridium-based ionic liquid has a similar structure to imidazolium, and its anion part is easier to change [19]. The presence of an aromatic group in this ionic liquid tends to stabilize the lipase structure and conformation so lipase can maintain its activity. Molecular dynamics simulations are usually used to better understand molecule interactions in the system. Using different solvents for the same protein can affect the stability of the protein. This happens due to the interaction between the solvent and the protein itself [20]. When a cosolvent system is used, we can know which solvent interacts more with the protein and affects its stability [21]. In this study, pyridinium-based ionic liquid was used in the hydrolytic reaction of lipase, which has not been widely reported in previous studies. This ionic liquid can be an alternative to imidazolium-based ionic liquids. There were four ionic liquids used in this study as reaction media for lipase, i.e. N-butylpyridinium tetrafluoroborate [C4Py]BF_4_, N-hexylpyridinium tetrafluoroborate [C6Py]BF_4_, N-octylpyridinium tetrafluoroborate [C8Py]BF_4_, and N-hexylpyridinium bromide [C6Py]Br. 4-Nitrophenyl acetate (pNPA) and 4-nitrophenyl palmitate (pNPP) were used as substrates for lipase hydrolytic activity. The use of these pyridinium-based ionic liquids as reaction media for lipase will give more information about how pyridinium-based ionic liquids affect lipase in terms of their hydrolytic activity.

## 2. Methods

### 2.1. Materials

Lipase from *Candida rugosa* (type VII, ≥700 unit/mg solid), 1-bromobutane, 1-bromohexane, 1-bromooctane, pyridine, 4-nitrophenyl acetate, and 4-nitrophenyl palmitate were purchased from Sigma Aldrich and used without further treatment. Ionic liquids were synthesized from 1-bromobutane, 1-bromohexane, 1-bromooctane, and pyridine. The hydrolytic activity of lipase was tested by using 4-nitrophenyl acetate and 4-nitrophenyl palmitate as substrates. Other chemical compounds used in this study were purchased from commercial sources and used without further purification.

### 2.2. Instrument

A digital scale from Mettler Toledo^®^ New Classic MF was used to weigh the reactant and reaction product. NMR spectrophotometer from AGILENT (500 MHz) was used for ionic liquids characterization. pH meter from Mettler Toledo^®^ was used to measure the pH from the buffer solution. A digital water bath from Benchmark MyBath was used to incubate the lipase and substrate for the lipase hydrolytic activity test. UV-Vis spectrophotometer from Genesys-10S was used to measure absorbance for the lipase hydrolytic activity test.

### 2.3. Ionic liquid synthesis

#### 2.3.1. General procedure for the synthesis of N-alkylpyridinium bromide

The ionic liquid used in this study was synthesized using the reported method [22]. Pyridine (0.5 mol) and alkyl bromide (0.5 mol) were added into a round bottom flask and refluxed for 72 h at 70 °C. The unreacted reactants were removed by washing them with ethyl acetate repeatedly. Ethyl acetate residue was then removed using a rotary vacuum evaporator, and the product was dried at room temperature (28–30 °C) to obtain a yellow–brown solid or thick solution [23]. The summary of spectroscopic data for N-butylpyridinium bromide, N-hexylpyridinium bromide, and N-octylpyridinium bromide is as follows:

***N*-butylpyridinium bromide [C_4_Py]Br:** ^1^H NMR (500 MHz, CDCl_3_) δ 9.51 (d, *J* = 5.95 Hz, 2H), 8.54 (t, *J* = 7.88 Hz, 1H), 8.15 (t, *J* = 7 Hz, 2H), 4.97 (t, *J* = 7.5 Hz, 2H), 2.00 (m, 2H), 1.37 (m, *J* = 7.65 Hz, 2H), 0.92 (t, *J* = 7.4 Hz, 3H).

***N*-hexylpyridinium bromide [C_6_Py]Br:** ^1^H NMR (500 MHz, CDCl_3_) δ 9.51 (d, *J* = 5.6 Hz, 2H), 8.52 (s, 1H), 8.14 (t, *J* = 7.15 Hz, 2H), 4.95 (t, *J* = 7.55 Hz, 2H), 2.01 (m, 2H), 1.27 (m, *J* = 3.3 Hz, 2H), 0.80 (t, *J* = 6.85 Hz, 3H).

***N*-octylpyridinium bromide [C_8_Py]Br:** ^1^H NMR (500 MHz, CDCl_3_) δ 9.48 (d, *J* = 5.25 Hz, 2H), 8.51 (s, 1H), 8.14 (t, *J* = 6.85 Hz, 2H), 4.93 (t, *J* = 7.5 Hz, 2H), 1.99 (m, 2H), 1.23 (m, *J* = 7.2 Hz, 2H), 0.78 (t, *J* = 6.5 Hz, 3H).

#### 2.3.2. General procedure for the synthesis of N-alkylpyridinium tetrafluoroborate

*N*-alkylpyridinium bromide was dissolved in acetone (0.05 mol *N*-alkylpyridinium in 20 mL acetone), and then sodium tetrafluoroborate was added with a molar ratio of 1:1. Subsequently, the mixture was stirred for 12 h at 25 °C and filtered to obtain the product. The acetone residue was evaporated using an evaporator to obtain *N*-alkylpyridinium tetrafluoroborate. The ionic liquid obtained from the synthesis was characterized using ^1^H-NMR. The summary of spectroscopic data for *N*-butylpyridinium tetrafluoroborate, *N*-hexylpyridinium tetrafluoroborate, and *N*-octylpyridinium tetrafluoroborate is as follows:

***N*-butylpyridinium tetrafluoroborate [C_4_Py][BF_4_]:** ^1^H NMR (500 MHz, CDCl_3_) δ 9.01 (d, *J* = 6.05 Hz, 2H), 8.49 (q, *J* = 7.3 Hz, 1H), 8.08 (q, *J* = 7 Hz, 2H), 4.68 (t, *J* = 7.6 Hz, 2H), 1.91–2.02 (m, 2H), 1.29–1.42 (m, 2H), 0.82 (s, 3H).

***N*-hexylpyridinium tetrafluoroborate [C_6_Py][BF_4_]:** ^1^H NMR (500 MHz, CDCl_3_) δ 9.01 (d, *J* = 5.75 Hz, 2H), 8.49 (t, *J* = 7.85 Hz, 1H), 8.07 (t, *J* = 9.7 Hz, 2H), 4.70 (t, *J* = 7.6 Hz, 2H), 1.95–2.01 (m, 2H), 1.24–1.33 (m, 2H), 0.82 (t, *J* = 6.95 Hz, 3H).

***N*-octylpyridinium tetrafluoroborate [C_8_Py][BF_4_]:** ^1^H NMR (500 MHz, CDCl_3_) δ 9.00 (d, *J* = 5.8 Hz, 2H), 8.48 (t, *J* = 7.8 Hz, 1H), 8.06 (t, *J* = 7 Hz, 2H), 4.69 (t, *J* = 7.55 Hz, 2H), 1.94–2.13 (m, 2H), 1.16–1.32 (m, 2H), 0.80 (t, *J* = 6.45 Hz, 3H).

### 2.4. Lipase hydrolytic activity test

The specific activity of lipase is defined as the amount of μmol product (4-nitrophenol) produced by lipase per minute per mg of enzyme and expressed in unit/mg. The hydrolytic activity of lipase was tested by adding the enzyme solution (enzyme in buffer solution) to the substrate mixture (substrate/ethanol/buffer = 1:4:95) and incubating for 10 min at various temperatures (40–60 °C). The incubation time was determined by how significant the absorbance value increased after some time. After 10 min of incubation, the absorbance value showed no significant increase. The buffer solution used in this study was set at pH 7. Lipase activity was calculated based on the absorbance of the mixture at 405 nm wavelength, which was an optimum wavelength for 4-nitrophenol, using a UV–Vis spectrophotometer. The absorbance values obtained were converted to a total product that formed from the reaction [24].

To study the effect of methanol on lipase hydrolytic activity, lipase was dissolved in methanol and buffer solution in various methanol concentrations (%) (v/v). The effect of ionic liquids was observed when ionic liquids were used as cosolvents of methanol. The ratios of methanol and ionic liquids varied from 10:0 to 10:5 (v/v). Specific activity from lipase was calculated using Equations (1) and (2):


(1) $$Activity(\frac{Unit}{mL})=\frac{(\text{A}-\text{i})\times Vt}{s\times Mw\;pNP\times t\times Ve}$$

(2) $$Specific\;Activity\;(\frac{Unit}{mg})=\frac{Activity\;(Unit/mL)}{Enzyme\;concentration\;(\frac{mg}{mL})},$$

where A represents the mixture absorbance at 405-nm wavelength, *i* the intercept value from 4-nitrophenol calibration curve, s the mean value from 4-nitrophenol calibration curve, *Vt* the total volume of the mixture (mL), *Ve* the enzyme volume (mL), *t* the time (min) for incubation, and *Mw pNP* the molar mass of 4-nitrophenol (139 μg/μmol). The unit used in this study is a relative activity that can be obtained from one specific activity compared to a control-specific activity.

### 2.5. Molecular dynamic study

*Candida rugosa* lipase crystal structure (PDB ID: 1TRH) was used as an initial structure for MD simulations. The MD simulations were conducted using GROMACS 2018.2 package and GROMOS 54a7 force field. SPC water model was used as water molecules in the MD simulation. An automated force field topology builder was used as a tool to generate ionic liquid force field. Lipase was solvated to solvents in cubic boxes with 90.38 × 90.38 × 90.38 Å dimensions. The total charges for each system were neutralized using sodium ions. Except for the water system, the other system was prepared using Packmol to ensure the amount of each molecule. The steepest descent minimization was conducted until the maximum force of the system was less than 10.0 kJ/mol. The equilibration was performed using NVT and NPT ensemble for every 100 ps with a 2-fs time step [21]. MD production was conducted using the modified Berendsen thermostat and Berendsen barostat [25]. The nonbonded forces were cut off at 12 Å, and particle mesh Ewald was applied to estimate the electronic interactions [26]. LINCS algorithms were used to constrain the bonds to H [27]. MD production was conducted for 50 ns for each system. The visualization was generated using VMD [28].

## 3. Results and discussion

### 3.1. Ionic liquid properties

The result of ^1^H-NMR confirmed that the ionic liquids were successfully synthesized. The most important wavenumber from ^1^H-NMR was found at around 4.9 ppm for *N*-alkylpyridinium bromide and 4.7 ppm for *N*-alkylpyridinium tetrafluoroborate. The wavenumbers around the range show the proton that is bound to the C atom is located between the aromatic group and alkyl group from ionic liquids. If the synthesis was not done successfully, then a wavenumber around that range would not exist. The physical properties for [C4Py]Br were a yellow-brown solid with 90.92% yield, [C6Py]Br was a thick yellow-brown solution with 95.09% yield, and [C8Py]Br was a thick yellow-brown solution with 71.22% yield. After a metathesis reaction between *N*-alkylpyridinium bromide and sodium tetrafluoroborate then, *N*-alkylpyridinium tetrafluoroborate formed. [C4Py]BF_4_ was a yellow solution with a 67.64% yield, [C6Py]BF_4_ was a yellow solution with a 56.21% yield, and [C4Py]BF_4_ was a yellow solution with a 60.21% yield.

### 3.2. Ionic liquid effect on lipase hydrolytic activity

**Figure 1:**
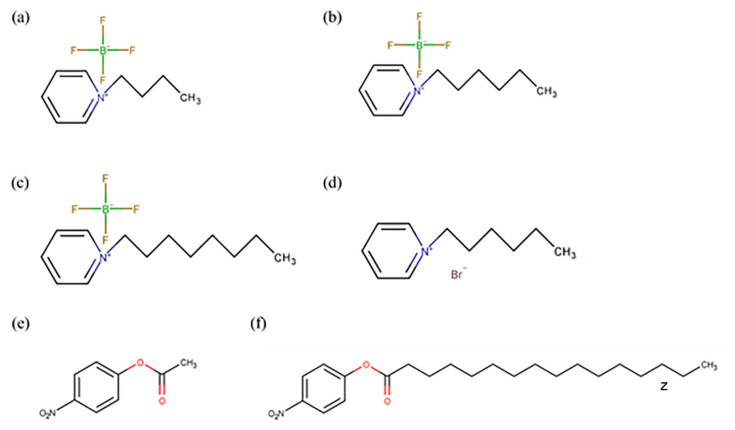
Structure of (a) [C4Py]BF_4_ ionic liquid, (b) [C6Py]BF4 ionic liquid, (c) [C8Py]BF_4_ ionic liquid, (d) [C6Py]Br ionic liquid, (e) pNPA, and (f) pNPP.

[C4Py]BF_4_, [C6Py]BF_4_, [C8Py]BF_4_, and [C6Py]Br ionic liquids are soluble in water solvents. These ionic liquids were used as reaction media for lipase by mixing it with water solvents. The effect of ionic liquids addition on the hydrolytic activity of *Candida rugosa* lipase was compared by using different substrates (pNPA and pNPP). The addition of an ionic liquid to water solvent did not significantly change the lipase hydrolytic activity (Figure 1). The highest lipase hydrolytic activity in the presence of ionic liquid was obtained by the addition of [C6Py]Br ionic liquid in the hydrolysis of the pNPP substrate. The addition of [C6Py]Br ionic liquids to water solvent could increase the hydrolytic activity of lipase by about 5.54%. Meanwhile, the lowest hydrolytic activity was obtained when [C4Py]BF_4_ ionic liquid was added to the water solvent to hydrolyze the pNPA substrate. The results show that the ionic liquid used in this study could not significantly increase the lipase hydrolytic activity. The presence of the ionic liquids did not damage or denaturize lipase. If lipase was denatured or the catalytic site was damaged because of the existence of the ionic liquid, then the lipase could not hydrolyze the substrate. Thus, the relative activity of lipase would be 0%.

**Figure 2:**
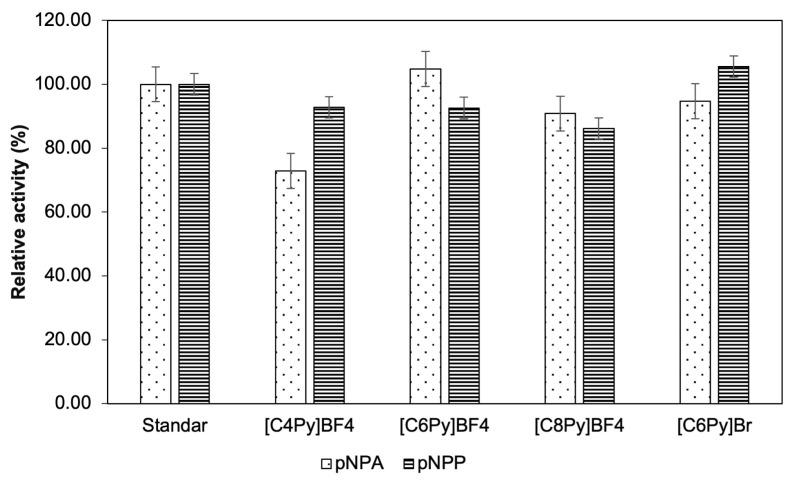
Effect of ionic liquid to lipase hydrolytic activity from *Candida rugosa* in water solvent at 55 °C.

Figure 1 is in line with a study conducted by Mohile in 2004 (Mohile et al., 2004). In this present study, [C6Py]BF_4_ provided a higher hydrolytic activity than [C4Py]BF_4_ [29]. This is similar to Mohile’s study, where [hmim]BF_4_ gave a higher lipase activity value than [bmim]BF_4_ [30]. Although the cations were from different lead compounds, using six-chain alkyls as cations could provide higher lipase activity than using four-chain alkyls. It is due to the hydrophobic properties that are found in [hmim]BF_4_ can stabilize lipase better than hydrophilic properties from [bmim]BF_4_. This occurred because the substrate used in this study has a higher similarity to [C4Py]BF_4_ than to [C6Py]BF_4_; thus, the ionic liquid can act as a competitive inhibitor of the substrate and then decrease lipase hydrolytic activity (Figure 1). The significant difference in the relative activity of Figure 2 was analyzed by using ANOVA. It is found that the p-value for the pNPA substrate is 5.87 × 10^−5^. This value is lower than 0.5; hence, the relative activity of each ionic liquid is significantly different. Meanwhile, the p-value for pNPP substrate is 0.129, which is also lower than 0.5. It shows that the relative activity of each ionic liquid is significantly different. Therefore, [C6Py]BF_4_ and [C6Py]Br were chosen for further study.

In this study, two different anions were used for the ionic liquids, i.e. bromide and tetrafluoroborate. This was done to study the effect of different anions on lipase hydrolytic activity. In 2013, Na et al. (2013) used imidazolium as the lead compound for cations from ionic liquids and studied its effect on lipase hydrolytic activity. From this study, different anions can have different effects on lipase hydrolytic activity [13]. The use of bromide as an anion can provide higher activity than that using tetrafluoroborate as an anion since, according to the Hofmeister series, bromide anion can strengthen hydrophobic interaction in protein better than tetrafluoroborate anion. As shown in Figure 1, the use of bromide anions for pyridinium-based ionic liquids also provided a higher hydrolytic activity value than that obtained using tetrafluoroborate anions. This implies that the different lead compounds of cation and anion of the ionic liquid provided the same pattern of lipase hydrolytic activity.

### 3.3. Effect of ionic liquid concentration on lipase hydrolytic activity

**Figure 3:**
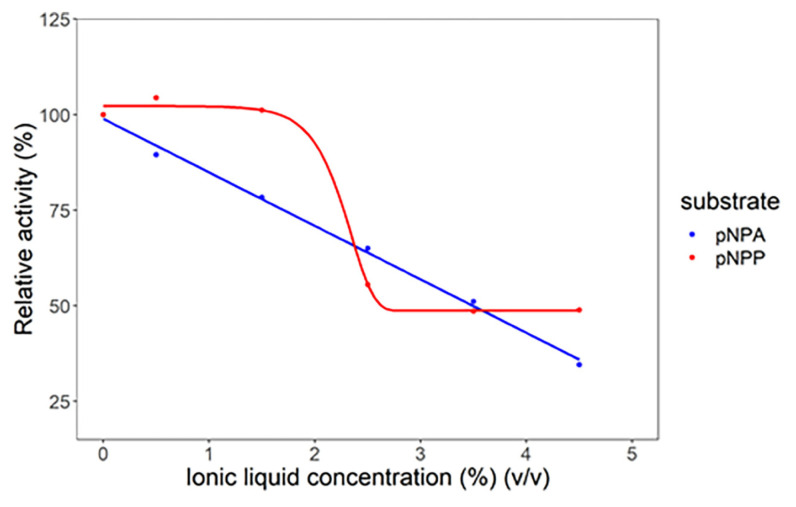
Effect of variations in the concentration of ionic liquids on the hydrolytic activity of *Candida rugosa* lipase at 55 °C.

The effect of the ionic liquid concentration added to the reaction media was investigated to determine the maximum concentration of ionic liquid to achieve the optimum lipase hydrolytic activity. Figure 3 illustrates how ionic liquid concentration can have a different effect on lipase hydrolytic activity, depending on the substrate. As shown in Figure 3, for the hydrolysis reaction of the pNPA substrate, the addition of [C6Py]BF_4_ ionic liquid tended to reduce the lipase hydrolytic activity, even if it was only at a concentration of 0.5%. This implies that the addition of [C6Py]BF_4_ ionic liquid can only be performed at a very small concentration (<0.5%) for lipase to hydrolyze the pNPA substrate. As for the pNPP substrate, [C6Py]Br, the ionic liquid can be added to a concentration of 1.5%. These results provide insights into what maximum ionic liquid concentration can be added to the lipase reaction media to hydrolyze the pNPA and pNPP substrates. From the results of the data fitting (Figure 3) using the Gompertz curve for the pNPP substrate, it was found that the ionic liquid has an inhibitory effect on the pNPP substrate using Equation (3).


(3) y=c+(d-c)×exp {-exp [b×(x-e)]}

where *c* is 48.72, *d* 102.21, *e* 2.35, and *b* 4.67, and the relative activity data for lipase form a sigmoid curve. This curve explains that a lesser concentration of ionic liquid used in the reaction will not reduce the relative activity of lipase, but when the amount of ionic liquid added to the reaction exceeds the concentration, the ionic liquids will inhibit the substrate and reduce the relative activity of lipase. As shown in Figure 2, the pNPP structure is more similar to the ionic liquids used in the experiment than pNPA. Thus, this is how the ionic liquids become competitive inhibitors for the pNPP substrate.

The ionic liquid concentration is an important factor to determine when using ionic liquids as the reaction media for lipases. A previous study has reported that the addition of ionic liquids may also decrease lipase hydrolytic activity. It was confirmed that the lipase hydrolytic activity was significantly reduced when the ionic liquid concentration exceeded a certain concentration. This occurred due to the formation of an aggregation of the ionic liquid that was added to the reaction media [13]. When the concentration of ionic liquid added to the reaction medium was lower than the critical micelle concentration (CMC), the ionic liquid could increase the lipase hydrolytic activity. However, if the ionic liquid concentration added to the reaction medium was higher than the CMC value, the lipase hydrolytic activity decreased [13].

### 3.4. Methanol effect on lipase hydrolytic activity

**Figure 4:**
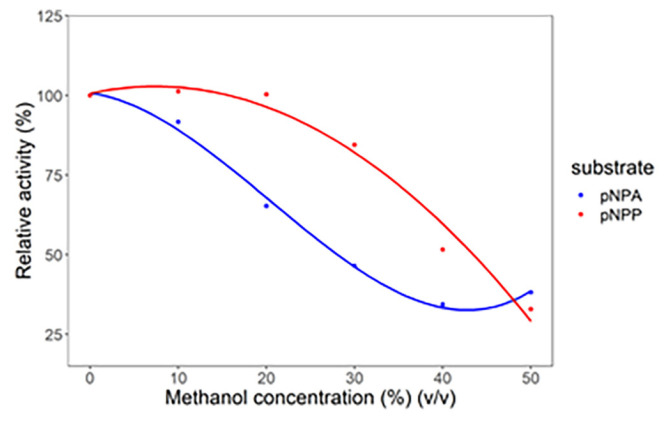
Effect of adding methanol to the hydrolytic activity of the *Candida rugosa* lipase.

Methanol was added to the water solvent to study the effect of organic solvents on lipase hydrolytic activity. Figure 4 shows how methanol can decrease lipase hydrolytic activity. As shown in Figure 4, the lipase-catalyzed hydrolysis of the pNPA with the addition of 10% methanol from the total volume of the reaction media was reduced. Moreover, for the hydrolysis of pNPP, the addition of methanol by 20% of the total volume of the reaction medium could still increase the lipase hydrolytic activity. These results indicate that *Candida rugosa* lipase provides a better activity value in hydrolyzing the pNPP substrate than pNPA when methanol is added to the reaction media.

Several ionic liquids reduce the lipase hydrolytic activity (Figures 1 and 3). Meanwhile, the addition of methanol could lower the lipase hydrolytic activity compared to the addition of ionic liquids (Figure 4). It proves that ionic liquids are better to use than methanol. These results are in accordance with the research conducted by Juneidi et al. (2010) [22]. When methanol was used as a reaction media, a decrease in the relative activity of lipase by more than 50% was observed, whereas when the ionic liquid was used as a reaction media, the activity of lipase was increased or decreased by 0%–25%, depending on the concentration of the added ionic liquid. The decrease in the lipase hydrolytic activity was caused by the addition of methanol provided an opportunity for the ionic liquid to act as a cosolvent of methanol. Methanol is needed in the lipase reaction, but at the same time, methanol can denaturize lipase itself. By using ionic liquids, the denaturation effect of methanol can be decreased. [C6Py]Br ionic liquid had the highest increase in lipase hydrolytic activity when the pNPP substrate was used (Figures 1 and 3). Therefore, [C6Py]Br ionic liquid was chosen as a cosolvent of methanol as a lipase reaction media to hydrolyze the pNPP substrate.

### 3.5. Ionic liquid as a cosolvent of methanol

**Figure 5:**
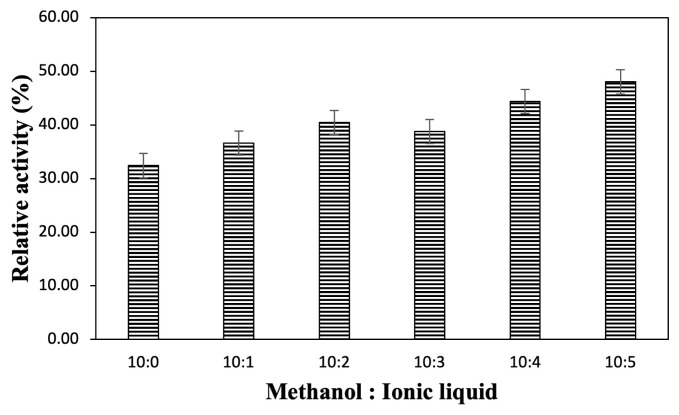
Effect of ionic liquid as a cosolvent of methanol on the hydrolytic activity of the *Candida rugosa* lipase.

The addition of ionic liquid as a cosolvent of methanol was predicted to reduce the denaturation effect of methanol on lipase. Figure 5 illustrates the effect of ionic liquid as the cosolvent of methanol. Based on Figure 5, the best ratio of methanol and [C6Py]Br ionic liquid was 10:5 (v/v). The addition of [C6Py]Br ionic liquids to the lipase reaction media as a cosolvent of methanol could increase the lipase hydrolytic activity by 15.61% than without ionic liquids. It can be concluded that ionic liquid can maintain the structure of lipase and reduce the denaturation effect of methanol.

Based on the research conducted by Pan et al. (2010) and Contesini and Carvalho (2006) [23, 24], using a mixture of organic solvents and ionic liquids as reaction media showed a better effect on lipase hydrolytic activity than using it separately. The use of pyridinium-based ionic liquids has the same trend as imidazolium-based ionic liquids in terms of increasing lipase hydrolytic activity.

### 3.6. Molecular dynamic study

Based on the experimental research, pyridinium-based ionic liquid with tetrafluoroborate as an anion could not increase the lipase hydrolytic activity when used as a methanol cosolvent. Conversely, using bromide as an anion for pyridinium-based ionic liquid could increase the lipase hydrolytic activity in methanol. Molecular dynamics methods can provide information about the differences between the two anions and have been proven to become a tool that can simulate the system’s stability that includes amino acids in the system [31].

**Figure 6:**
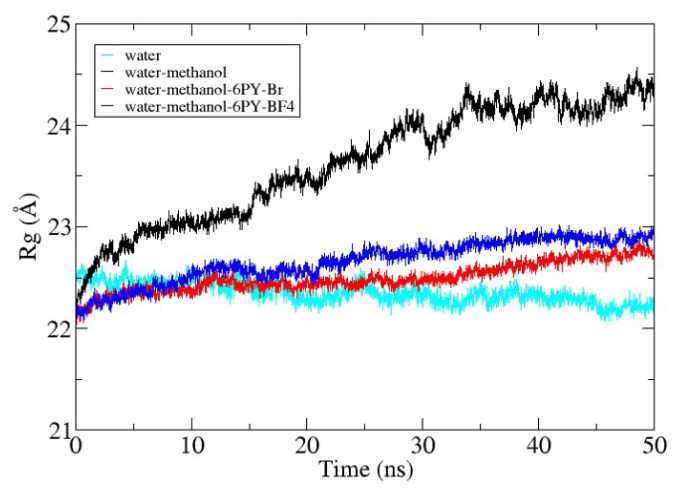
The radius of gyration from 50-ns production of *Candida rugosa* lipase.

**Figure 7:**
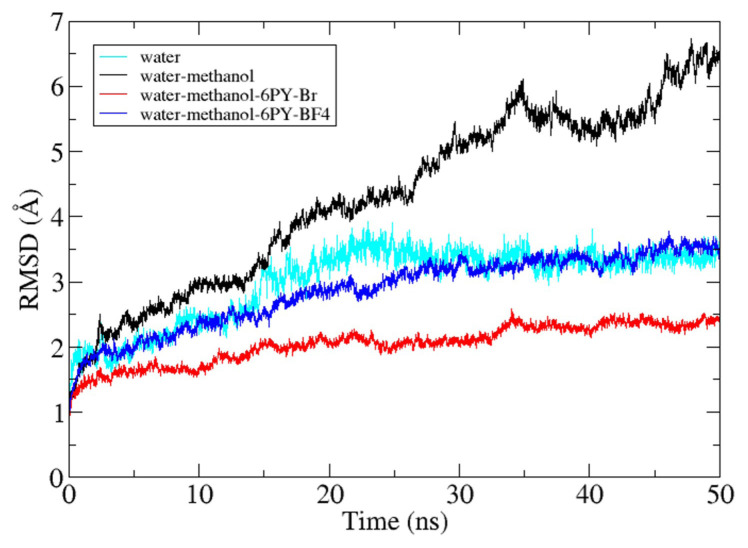
Backbone RMSD of *Candida rugosa* lipase for each system.

Figure 6 shows the radius of gyration for lipase during 50 ns of simulation. The radius of gyration (Rg) of lipase for each system has its own pattern. When the water system was used as a reference, the Rg value of the lipase in the methanol-water system was higher than the Rg of lipase in the ionic liquid–methanol-water system. The Rg value of lipase for each system at the end of the simulation was 22.21 Å for the water system, 24.44 Å for the water-methanol system, 22.75 for the water-methanol–[C6Py]Br system, and 22.93 Å for the water-methanol–[C6Py]BF_4_ system. The increase in Rg value was caused by the interaction between methanol and the residue on the outer lipase surface. Methanol disrupted and weakened the noncovalent interaction between residues so that the distance between the residues was further away and increased the Rg value of the lipase. In this case, both ionic liquids help in reducing the denaturation effect of methanol so that the Rg in the ionic liquid system does not increase significantly [32]. The further study of the lipase structure during the simulation, the root mean squared deviation or displacement (RMSD) value of the lipase was analyzed in each system. The result in Figure 7 shows that the Rg and RMSD values have similar results. Differences were observed in the Rg and RMSD values of lipase in the water and water-methanol–[C6Py]Br systems. The Rg value of lipase in the water-methanol–[C6Py]Br system was higher than the Rg value of lipase in the water system, whereas the RMSD value of lipase was reversed. This occurred because lipase in the water system has a tendency to make its structure more compact. In this case, lipase from *Candida rugosa* tends to move to its closed form. In contrast to the water system, lipase in the water-methanol–[C6Py]Br system maintains the stability of the conformation, thereby increasing the Rg value but decreasing the RMSD value. From these results, it can be concluded that the use of [C6Py]Br ionic liquid as a cosolvent of methanol can maintain the active conformation of lipase and reduce the denaturation effect of methanol.

As shown in Figures 6 and 7, lipase in the water-methanol system always gives higher Rg and RMSD values than lipases in other systems. This was in accordance with the research conducted by Mohtashami et al. (2019), who studied lipase from *Burkholderia cepacia* (BCL) in water and methanol systems. When the amount of methanol in the system increases, the Rg and RMSD values of BCL also increase [33]. This helps in understanding how methanol can have a denaturing effect on lipases so that their Rg and RMSD values increase.

**Figure 8:**
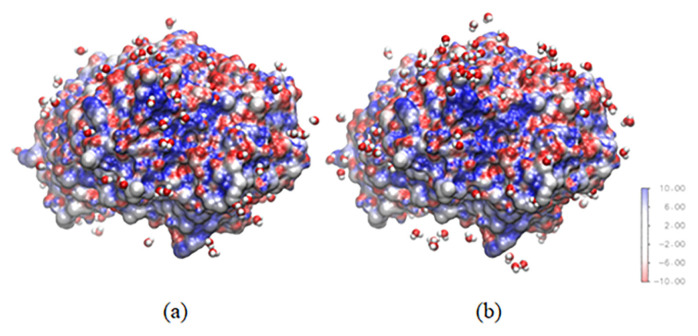
Water within 3 Å of any protein atom in the (a) water-methanol–[C6PY]Br and (b) water-methanol–[C6PY]BF4 systems.

The water around lipase can show how lipase tends to catalyze the reaction. Lipases have two activities depending on the amount of water around their surfaces. When the water around the lipase surface is sufficient, lipase can have hydrolytic activity; otherwise, lipase will catalyze an esterification reaction. Both systems have the same amount of water around lipase (Figure 8), which implies that both lipases in different systems will have the same activity. In agreement with the experiment study, lipase in [C6Py]BF_4_ and [C6Py]Br ionic liquids have the same value for the specific activity.

**Figure 9:**
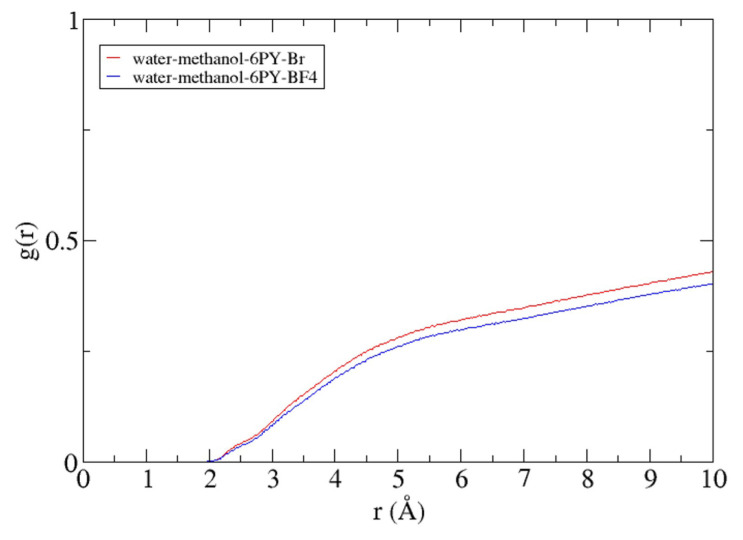
RDF of each cation from the system with the reference point using protein masses.

**Figure 10:**
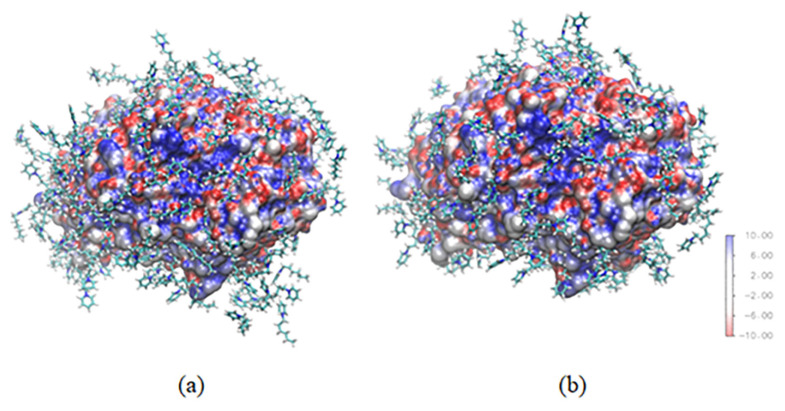
N-hexylpyridinium within 3 Å of any protein atom in the (a) water-methanol–[6PY]Br and (b) water-methanol–[6PY]BF4 systems.

The advantage of using the molecular dynamics method to study the structure of lipases was notable in observing interactions among molecules. Radial distribution function (RDF) analysis could monitor the interaction between solvent and lipase. As shown in Figure 9, the cationic part of the pyridinium-based ionic liquid does not interact closely with the lipase. This is the case for both ionic liquids. At a radius of less than 2 Å from the center of mass of the lipase, no cations from the two ionic liquids were present. The cationic part of the ionic liquid was discovered after a distance of 2 Å and continued to increase. This indicates that the cationic part of the ionic liquid only acts as a part of the solvent and gives effect to lipase only through long-range interactions. Figure 10 illustrates how the cation (N-hexylpyridinium) from the ionic liquids interacts with the lipase surface. The cation that surrounded the lipase within 3 Å did not have a significantly different amount (142 molecules for water–methanol–[6PY]Br system and 112 molecules for water–methanol–[6PY]BF_4_ system). This is in agreement with the result shown in Figure 9, where the amount of cation in the water-methanol–[6PY] Br system (red) has slightly higher molecules.

**Figure 11:**
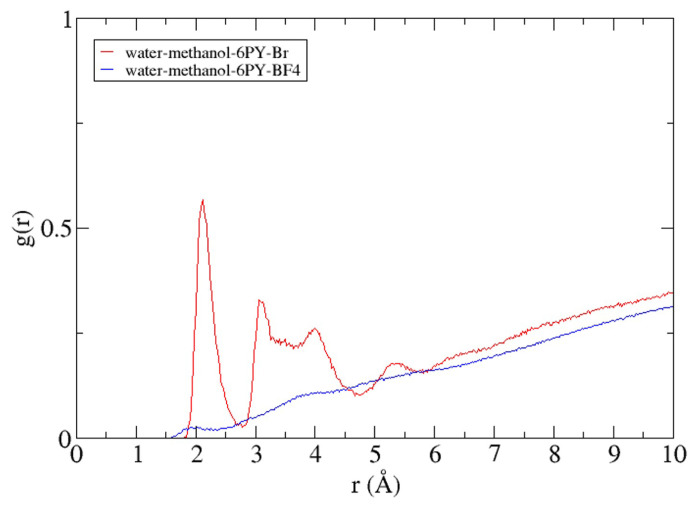
RDF of each anion from the system with reference point using protein masses.

As shown in Figure 11, different anions give different RDF curve patterns. The tetrafluoroborate anion of the [C6Py] BF_4_ ionic liquids cannot be found before a radius of 1.52 Å from the center of mass of the lipase and does not form clusters or layers around the lipase. A different curve pattern can be found on the RDF curve for the bromide anion of the [C6Py] Br ionic liquid. Bromide anions can be found at a radius of 1.8 Å and have a peak at a distance of 2.1 Å. At this point, the highest amount of bromide anion was found around the lipase and formed the first layer around the lipase. These results show the bromide ion as an anion can provide a short-range interaction with the residues on the outer lipase surface, especially the positively charged residue. Lipase can maintain its stability and reduce the denaturation effect of methanol using a short-range interaction between lipase and the anion of the [C6Py]Br ionic liquid. These results also agree with the Rg (Figure 6) and RMSD (Figure 7) values of the lipase.

**Figure 12:**
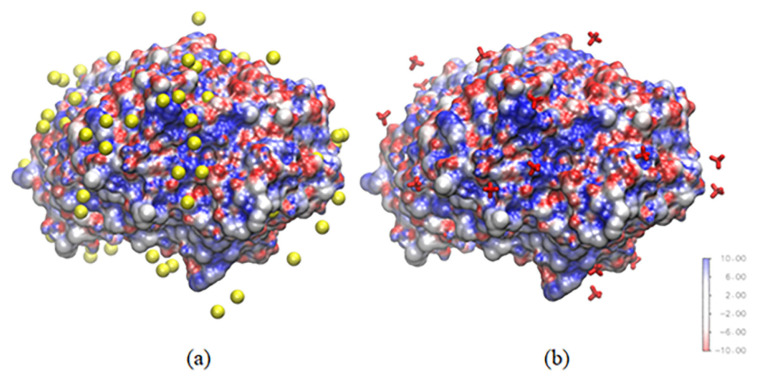
Anion within 3 Å of any protein atom in the (a) water-methanol–[6PY]Br and (b) water-methanol–[6PY]BF_4_ systems.

The RDF value can be represented with some snapshots from the simulation. Figure 12 shows a snapshot from the last frame of the simulation for the two different systems. Within 3 Å of any protein atom, 67 bromide atoms can be found surrounding the lipase, whereas 22 tetrafluoroborate molecules can be found in the other system. The amount of bromide that can be found within 3 Å of protein is three times higher than the amount of tetrafluoroborate. This showed how the anion from [C6Py]Br ionic liquid gave more effect to stabilize the lipase using a short-range interaction between the anion and positively charged lipase surface.

**Figure 13:**
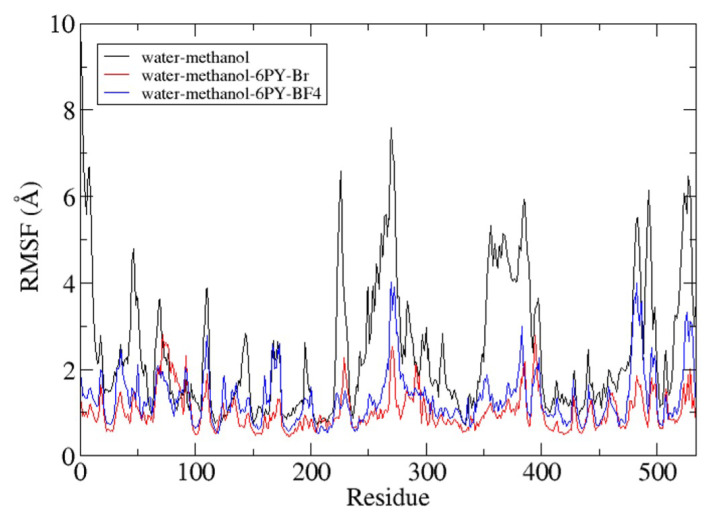
Backbone RMSF of *Candida rugosa* lipase during 50-ns simulation.

The root means square fluctuation (RMSF) value of each lipase residue can provide information about the stability of each residue in the lipase. As shown in Figure 13, lipase in the water-methanol system has the highest fluctuating RMSF value, especially the residue, which has a turn and coil secondary structure. The use of ionic liquid as a cosolvent of methanol can reduce the fluctuation of each residue during the simulation. Although the two ionic liquids can help decrease the fluctuation of the residues, [C6Py]Br ionic liquid provides higher stability than [C6Py]BF_4_ ionic liquid. This happens according to the results in Figure 10, showing that the anion of [C6Py]Br can provide the short-range interaction that stabilizes the residues of lipase.

These results provide the same pattern as the imidazolium-based ionic liquid previously studied by Burney and Pfaendtner (2013) [34]. The research studied the stability of the *Candida rugosa* lipase structure in water, octane, and imidazolium-based ionic liquids. Based on the results of Burney and Pfaendtner (2013), the imidazolium-based ionic liquids provide the highest stability for lipase from *Candida rugosa*. The use of ionic liquids can reduce the fluctuation of the residues in lipase. The result of this study shows that the [C6Py]Br ionic liquid can maintain the stability of *Candida rugosa* lipase when it is used as a cosolvent of methanol both experimentally and using the molecular dynamics approach.

**Figure 14:**
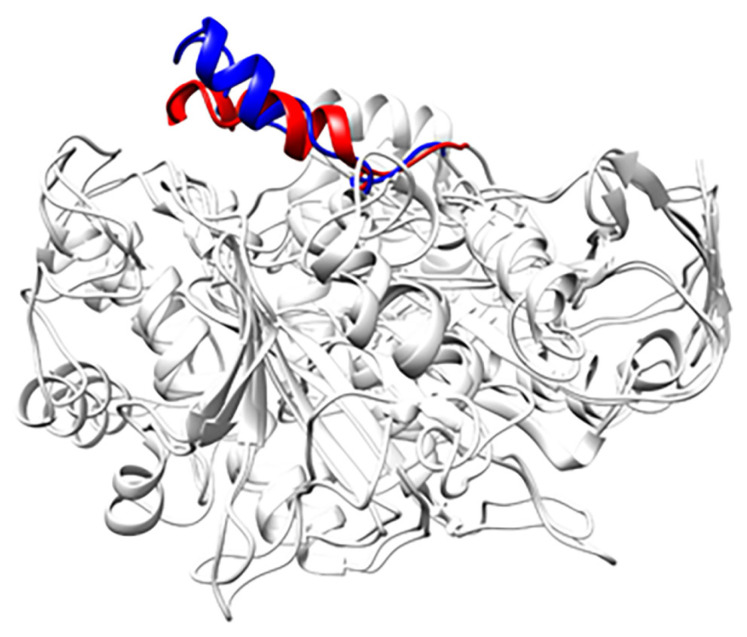
Lid position in lipase between the two different systems (blue in water-methanol–[6PY]Br system and red in water-methanol–[6PY]BF_4_ system).

The lipase structure used in this study is *Candida rugosa* lipase in its closed conformation. We can use this to study how the reaction medium can affect the lid behavior in lipase. As shown in Figure 14, the lid from lipase in the water-methanol–[6PY]Br system moves farther from the protein core than the lid from lipase in the water-methanol–[6PY]BF_4_ system. The radius of gyration for lipase in the water-methanol–[6PY]Br and water-methanol–[6PY]BF_4_ systems is 22.75 and 22.93 Å, respectively. This lid behavior can make the substrate interact with the catalytic residue in the active sites much easier. This is one of the reasons why lipase in the water-methanol–[6PY]Br system has higher activity than lipase in the water-methanol–[6PY]BF_4_ system in the experimental study.

## 4. Conclusion

The present study aimed to synthesize pyridinium-based ionic liquids and examine their applications to improve lipase hydrolytic activity. Pyridinium-based ionic liquids as additional solvents in water solvents did not significantly increase the lipase hydrolytic activity for pnpa and pnpp substrates. Moreover, pyridinium-based ionic liquids did not reduce the lipase hydrolytic activity to an extreme. These findings suggest that, in general, the pyridinium-based ionic liquids can maintain the stability of the lipase when added to the water solvent for the lipase-catalyzed reaction. The use of [C6Py] Br as a cosolvent of methanol in the methanol–water reaction media was proven to increase the lipase hydrolytic activity by 15.61% more than those without ionic liquids. This is because the ionic liquid can maintain lipase stability and resist the denaturation effect from methanol.

Research using molecular dynamics simulation methods provides more information about the interaction between ionic liquids and lipase. The most important part of the pyridinium-based ionic liquid is the anion. In this study, the bromide anion stabilized the lipase better than the tetrafluoroborate anion. The results of the study using the molecular dynamics simulation method are also in accordance with the results of experimental research in which the pyridinium-based ionic liquid with bromide anion can increase the lipase hydrolytic activity higher than the pyridinium-based ionic liquid using tetrafluoroborate anion. The major limitation of this study is the anion variation. For further research, the use of anion should be varied to establish a greater degree of accuracy on this matter.

## Data Availability

The data that support the findings of this study are available from the corresponding author, RH, upon reasonable request.

## References

[1] Bhalerao MS Kulkarni AV Patwardhan, Ultrasound-assisted hemoenzymatic epoxidation of soybean oil by using lipase as biocatalyst, Ultrason Sonochem 2018 40 912 920 10.1016/j.ultsonch.2017.08.042 28946503

[2] Faber K 2011 Biotransformation in Organic Chemistry, A Textbook 6th ed Heidelberg Springer 2011

[3] Ülker S Özel A Çolak A Alpay Karaoğlu Ş Isolation, production, and characterization of an extracellular lipase from *Trichoderma harzianum* isolated from soil Turkish Journal of Biology 2011 35 543 550 10.3906/biy-1004-107

[4] Ananthi S Ramasubburayan R Palavesam A Immanuel G Optimization and purification of lipase through solid state fermentation by bacillus cereus MSU as isolated from the gut of a marine fish *Sardinella longiceps* International Journal of Pharmacy and Pharmaceutical Sciences 2014 6 291 298

[5] Priego J Ortíz-Nava C Carrillo-Morales M López-Munguía A Escalante J Solvent engineering: an effective tool to direct chemoselectivity in a lipase-catalyzed Michael addition Tetrahedron 2009 65 536 539 10.1016/j.tet.2008.10.103

[6] Guo J Chen CP Wang SG Huang XJ A convenient test for lipase activity in aqueous-based solutions Enzyme and Microbial Technology 2011 71 8 12 10.1016/j.enzmictec.2015.01.005 25765304

[7] Moniruzzaman M Nakashima K Kamiya N Goto M Recent advances of enzymatic reactions in ionic liquids Biochemical Engineering Journal 2010 48 295 314 10.1016/j.bej.2009.10.002

[8] Lozano P De Diego T Carrié D Vaultier M Iborra JL Over-stabilization of *Candida antarctica* lipase B by ionic liquids in ester synthesis Biotechnology Letters 2011 23 1529 1533

[9] Nessim MI Zaky MT Deyab MA Three new gemini ionic liquids: Synthesis, characterizations and anticorrosion applications Journal of Molecular Liquids 2018 266 703 710 10.1016/j.molliq.2018.07.001

[10] Vraneš M Tot A Janković N Gadžurić S What is the taste of vitamin-based ionic liquids? Journal of Molecular Liquids 2010 9 276 902 909 10.1016/j.molliq.2018.12.085

[11] Kaftzik N Wasserscheid P Kragl U Use of ionic liquids to increase the yield and enzyme stability in the β-galactosidase catalysed synthesis of N-acetyllactosamine Organic Process Research & Development 2002 6 553 557 10.1021/op0255231

[12] Lv S Zou X Qian H Qin J Jin Q Impact of ionic liquid properties on selective enrichment of glycerides in direct lipase-catalyzed esterification The Royal Society of Chemistry 2016 6 108697 108707 10.1039/c6ra24089e

[13] Na L Weiyan D Zhuonan H Wei Z Shoujiang W Effect of imidazolium ionic liquids on the hydrolytic activity of lipase Cuihua Xuebao/Chinese Journal of Catalysis 2013 34 769 780 10.1016/s1872-2067(11)60521-4

[14] Vidya P Chadha A The role of different anions in ionic liquids on Pseudomonas cepacia lipase catalyzed transesterification and hydrolysis Journal of Molecular Catalysis B: Enzymatic 2009 57 145 148 10.1016/j.molcatb.2008.08.007

[15] Barbosa MS Santos AJ Carvalho NB Figueiredo RT Pereira MM Enhanched activity of immobilized lipase by phosphonium-based ionic liquids used in the support preparation and immobilization process ACS Sustainable Chemistry & Engineering 2019 7 15648 15659 10.1021/acssuschemeng.9b03741

[16] Zhao H Toe C “Water-like” ammonium-based ionic liquids for lipase activation and enzymatic polymerization” Process Biochemistry 2020 98 59 64 10.1021/acsomega.9b02118

[17] Peng L Wang Z Zhu H Zeng T Zhou W Synthesis, physico-chemical properties of novel tropine-amino acid based ionic liquids and their effects on the lipase activity Journal of Molecular Liquids 2021 342 116938

[18] Crosthwaite JM Muldoon MJ Dixon JK Anderson JL Brennecke JF Phase transition and decomposition temperatures, heat capacities and viscosities of pyridinium ionic liquids The Journal of Chemical Thermodynamics 2005 37 559 568 10.1016/j.jct.2005.03.013

[19] Pieczyńska A Ofiarska A Borzyszkowska AF Białk-Bielińska A Stepnowski P A comparative study of electrochemical degradation of imidazolium and pyridinium ionic liquids: A reaction pathway and ecotoxicity evaluation Separation and Purification Technology 2015 156 522 534 10.1016/j.seppur.2015.10.045

[20] Rasoolidanesh M Astaraki M Mostafavi M Rezvani M Ganji MD Toward efficient enantioseparation of ibuprofen isomers using chiral BNNTs: Dispersion corrected DFT calculations and DFTB molecular dynamic simulations Diamond and Related Materials 2021 119 108561 10.1016/j.diamond.2021.108561

[21] Burney PR Pfaendtner J Structural and dynamic features of Candida rugosa lipase 1 in water, octane, toluene, and ionic liquids BMIM-PF6 and BMIM-NO3 The Journal of Physical Chemistry B 2013 117 2662 2670 10.1021/jp312299d 23387335

[22] Juneidi I Hayyan M Hashim MA Hayyan A Pure and aqueous deep eutectic solvents for a lipase-catalysed hydrolysis reaction Biochemical Engineering Journal 2017 117 129 138 10.1016/j.bej.2016.10.003

[23] Pan S Liu X Xie Y Yi Y Li C Esterification activity and conformation studies of Burkholderia cepacia lipase in conventional organic solvents, ionic liquids and their co-solvent mixture media Bioresource Technology 2010 101 9822 9824 10.1016/j.biortech.2010.07.107 20713309

[24] Jaeger K-E Dijkstra BW Reetz MT Bacterial biocatalysts: Molecular biology, three-dimensional structures, and biotechnological applications of lipases Annual Review of Microbiology 1999 53 315 351 10.1146/annurev.micro.53.1.315 10547694

[25] Berendsen HJC Postma JPM van Gunsteren WF Dinola WF Haak JR Molecular dynamics with coupling to an external bath The Journal of Chemical Physics 1984 81 3684 3690

[26] Darden T York D Pedersen L Particle mesh Ewald: an N-log(N) method for Ewald sums in large systems The Journal of Chemical Physics 1993 98 10089

[27] Hess B Bekker H Berendsen HJC Faraaije JGEM LINCS: A linear constraint solver for molecular simulations Journal of Computational Chemistry 1997 18 1463 1472

[28] Humphrey W Dalke A Schulten K VMD: Visual molecular dynamics Journal of MolecularGraphics 1996 14 33 38 10.1016/0263-7855(96)00018-5 8744570

[29] Mohile SS Potdar MK Harjani JR Nara SJ Salunkhe MM Ionic liquids: efficient additives for *Candida rugosa* lipase-catalysed enantioselective hydrolysis of butyl 2-(4-chlorophenoxy)propionate Journal of Molecular Catalysis B: Enzymatic 2004 30 185 188 10.1016/j.molcatb.2004.05.002

[30] Contesini FJ de Oliveira Carvalho P Esterification of (RS)-Ibuprofen by native and commercial lipases in a two-phase system containing ionic liquids Tetrahedron: Asymmetry 2006 17 2069 2073 10.1016/j.tetasy.2006.07.020

[31] Ganji MD Larijani HT Alamol-hoda R Mehdizadeh M First-principles and Molecular Dynamics simulation studies of functionalization of Au32 golden fullerene with amino acids Scientific Report 2018 8 11400 10.1038/s41598-018-29887-5 PMC6065410 30061669

[32] Herskovits TT Gadegbeku B Jaillet H On the structural stability and solvent denaturation of proteins Journal of Biological Chemistry 1970 245 2588 2598 10.1016/S0021-9258(18)63111-4 5445802

[33] Mohtashami M Fooladi J Haddad-Mashadrizeh A Housaindokht MR Monhemi H Molecular mechanism of enzyme tolerance against organic solvents: Insights from molecular dynamics simulation International Journal of Biological Macromolecules 2019 122 914 923 10.1016/j.ijbiomac.2018.10.172 30445665

[34] Burney PR Pfaendtner J Structural and dynamic features of *Candida rugosa* lipase 1 in water, octane, toluene, and ionic liquids BMIM-PF6 and BMIM-NO3 The Journal of Physical Chemistry B 2013 117 2662 2670 10.1021/jp312299d 23387335

